# Lip Bumper Therapy Does Not Influence the Sagittal Mandibular Incisor Position in a Retrospective CBCT Study

**DOI:** 10.3390/jcm11206032

**Published:** 2022-10-13

**Authors:** Olivia Griswold, Chenshuang Li, Justin C. Orr, Normand S. Boucher, Shalin R. Shah, Chun-Hsi Chung

**Affiliations:** 1 Department of Orthodontics, School of Dental Medicine, University of Pennsylvania, Philadelphia, PA 19104, USA; 2 Private Practice, Princeton Junction, West Windsor, NJ 08550, USA

**Keywords:** interceptive orthodontics, lip bumper, CBCT, mandibular incisor

## Abstract

Lip bumper (LB) therapy is used as a treatment approach for mild to moderate crowding without extraction of teeth. Previous studies demonstrated that LB increases arch length through molar uprighting and lateral expansion. However, the effects of LB on mandibular incisors are inconclusive. The controversial results from different studies may be due to limitations including absence of a control group and/or use of 2D radiography. To address this issue, the current retrospective longitudinal CBCT study compared a rapid maxillary expansion (RME) group with no lower treatment [16 patients (9 females, 7 males); median age 8.86 years at T1 and 11.82 years at T2] and an RME + LB group [18 patients (13 females, 5 males); median age 9.46 years at T1 and 12.10 years at T2]. The CBCTs taken before and after phase 1 treatment were 3D superimposed based on the mandibular structure and were measured to determine the angular and linear changes of the mandibular incisors over the course of LB treatment. For comparisons between different timepoints within a group, a Wilcoxon matched-pairs signed rank test was used. For intergroup comparisons, a Mann–Whitney *U* test was used. Both groups showed eruption and protrusion of the mandibular incisors during the observation period, while there was no significant change in proclination of the lower incisors. When comparing the discrepancy of change between groups, there was no statistically significant difference detected. In summary, by utilizing a longitudinal 3D database, the current study demonstrated that the effect of LB on the position of the mandibular incisors is limited.

## 1. Introduction

Lip bumpers are functional, removable devices that utilize the force of the circumoral musculature to cause tooth movement [1]. Lip bumpers can also be used as a habit-breaking appliance in patients with a lip-sucking habit [1]. According to the literature, a lip bumper has the effects of increasing arch circumference through lateral and anterior-posterior expansion [2,3,4]. Thus, a lip bumper (LB) in conjunction with rapid maxillary expansion (RME) is a common tool in the orthodontic armamentarium to gain arch circumference in cases with mild to moderate crowding, which offers an opportunity to gain the necessary space as an alternative to extraction treatment [4].

Regarding the specific effects of lip bumpers on the mandibular dentition, it has been reported to increase transverse arch width, cause labial movement of the lower incisors and molar uprighting [5,6,7,8,9,10,11]. The lip bumper appliance can be customized by adjusting the arch wire to provide more posterior expansion or lower incisor flaring [1]. In as early as the 1970s, Bergersen et al. stated 95% of lip bumper cases exhibit forward migration of the lower incisors and distal movement of the first molar [12]. Later, Grossen et al. had similar findings of mandibular incisor protrusion and proclination along with mandibular molar distal tipping [6]. O’Donnell et al. also reported that following one year of lip bumper therapy, there was proclination and protrusion of the lower incisors as well as distal tipping of the first and second molars [5]. In agreement with these findings, Davidovitch et al. performed a prospective controlled study and found that lower incisors in the lip bumper group had a six times greater change in inclination and significant distal crown tip of the first molars compared to the control group [7]. While Subtelny et al. found that the majority of cases treated with a lip bumper had molar uprighting and distal movement, contrary to much of the literature, only 44% of cases had lower incisor protrusion [13]. Jacob et al. found the distal tip of the mandibular first molars, lower incisor tip protrusion and for the first time, reported 1.5 mm of first molar and lower incisor vertical development [8]. Thus, the effects of lip bumpers on mandibular molars are consistent across all these studies, but the effect on mandibular incisors varies in different studies.

It is worth noting that, all prior literatures have only utilized 2D lateral cephalograms or lateral tomographs to provide cephalometric measurements and to analyze changes over the course of lip bumper treatment. Multiple studies lacked a control group [5,6,8], and in some studies, the lip bumper was not the only orthodontic device applied to the mandibular arch [12]. Lip bumpers are routinely used in early mixed dentition and during this age, a significant amount of mandibular dentoalveolar growth and development is expected. A control group that is age-matched without mandibular treatment allows for assessment of natural changes due to growth and development over an experimental treatment period. In the prior studies that lack a control group, we cannot distinguish if the changes observed over the course of LB therapy are outcomes due to treatment effect, expected growth and development, or a combination of both. In 2009 and 2020, systematic reviews were performed to determine the strength of the evidence regarding lip bumper treatment effects. The 2009 systematic review by Hashish et al. found one study that met the inclusion criteria [14]. The 2020 systematic review by Santana et al. included 6 studies, no meta-analysis was performed due to heterogeneity and it was determined that the level of certainty regarding present data on lip bumper therapy was low [15].

As the orthodontic field continues to increase its use of CBCT technology, the availability of new data continues to grow. CBCT images allow for more precise measurement of the dentoskeletal complex and visual access to measurements not previously observable on 2D radiography [16,17]. CBCT technology offers us the opportunity to reinvestigate previous findings without the distortion, overlap and magnification of formerly studied 2D images [17,18]. Therefore, the aims of this study are to evaluate the changes of the mandibular incisors in response to lip bumper therapy, to help clinicians better understand the treatment effects of the lip bumper and in turn, optimize treatment plans and outcomes in orthodontics.

## 2. Materials and Methods

The study was conducted in accordance with the Declaration of Helsinki, and approved by the Institutional Review Board of the University of Pennsylvania (protocol # 850683 and date of approval: 31 January 2022). The study is a retrospective longitudinal CBCT study utilizing data from two private practice orthodontic clinics. Two separate groups were studied. The inclusive criteria are: patients (1) presented with mixed dentition; (2) had all four lower incisors erupted at T1; (3) skeletal class I or mild class II (0° < ANB angle < 6°); (4) had RME or RME + LB as phase I orthodontic treatment; (5) had no orthodontic treatment prior to their T1 CBCT. The exclusive criteria are: patients (1) had craniofacial syndrome; (2) had history of trauma to craniofacial region; (3) had missing, impacted, large caries lesion, periapical lesion, ankylosis or trauma to mandibular incisors; (4) had orthodontic devices other than RME or LB delivered during the phase I treatment. The T2 CBCT was taken as the initial records for comprehensive phase II treatment (Table 1). The Voxel size of all the CBCT images was 0.400 mm × 0.400 mm × 0.400 mm.

The RME group was treated with bonded RME (with occlusal platforms covering all posterior teeth) and no mandibular arch treatment. The group contained 16 subjects, 9 females and 7 males. The median age at T1 was 8.86 years (range: 7.62 to 10.48 years). The median age at T2 was 11.82 years (range: 10.81 to 13.82 years). The CBCT images for this experimental group were taken a median of 2.98 years apart (range: 2.11 to 3.84 years).

The RME + LB group was treated with bonded RME for the maxilla and LB for the mandible. The group contained 18 subjects, 13 females and 5 males. The median age at T1 was 9.46 years (8.29 to 10.29 years). The median age at T2 was 12.10 years (10.98 to 12.99 years). The CBCT images for this experimental group were taken a median of 2.62 years apart (range: 1.75 to 3.66 years). Prefabricated CG lip bumpers (Dentsply GAC international, NY, USA) were used. The lip bumpers were activated transversely by expanding the wire facially to 1 mm wider than the buccal tubes on the mandibular first molars at every visit until the mandibular molars were fully uprighted. Anteriorly, the lip bumpers were adjusted each visit in order to ensure they were positioned in the middle-third and 2 mm labial to the facial surface of the lower incisors. The median length of time in lip bumper treatment was 1.79 years (range: 1.16 to 2.66 years).

The CBCT DICOM files were imported into Dolphin 3D software (Dolphin Imaging; version 11.95 Premium, Chatsworth, CA, USA) and oriented by using the Frankfort plane as the horizontal plane in the “Orientation” module. In addition, the orientation was adjusted axially so that the posterior borders of the orbits from a lateral view overlapped each other, and coronally so that the inferior borders of both orbits sat on the same plane from a frontal view. In the “Superimposition of Volumes” module of the Dolphin 3D software, the T1 and T2 CBCT images were superimposed according to American Board of Orthodontics (ABO) standards of mandibular superimposition and in accordance with methods followed by previous research studies [16,18].

**Figure 1:**
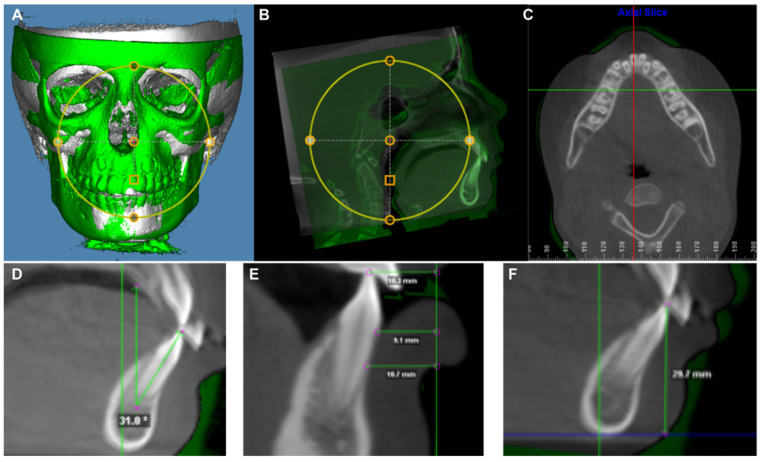
The demography of CBCT image analysis utilized in current study. (**A**) A screenshot of the superimposition of T1 and T2 CBCT images in a 3D reconstructed view. Th T1 CBCT image is represented in white and the T2 CBCT image is presented in green. (**B**) A screenshot of the superimposition of T1 and T2 CBCT images in midline sagittal slice view. The superimposition was performed based on the mandibular structure. The T1 CBCT image is represented in white and the T2 CBCT image is presented in green. (**C**) The axial slice was used to find the sagittal slice (red line) that was a best fit through the center of the mandibular central incisors. (**D**) Mandibular central incisor inclination measurement on the sagittal slice: Best fit line from tip of incisor through root apex measured to true vertical. (**E**) Mandibular central incisor protrusion measurement on the sagittal slice: The buccal surface of the tooth to true vertical line established at 10 mm ahead of the T1 symphysis (green vertical line). (**F**) Mandibular central incisor vertical position measurement on the sagittal slice: Tip of incisor to true horizontal line established at the T1 inferior border of symphysis (blue line).

Utilizing the superimposed CBCTs within the “Superimposition of Volumes” module, data collection of both linear measurements and angular measurements was completed using a sagittal slice that was a best fit through the center of the mandibular central incisors using coronal and axial views (Figure 1). The thickness of the sagittal slice was set to a 1-voxel slice.

The mandibular incisor inclination was measured as a best fit line through the incisal tip and apex to the true vertical of the superimposition. Buccally angulated measurements were deemed to be positive while lingually angulated measurements were deemed negative. Over the time period, a positive value was indicative of proclination while a negative value was indicative of retroclination.

The protrusion of the mandibular incisor was measured at three levels: incisor tip, cementoenamel junction (CEJ) and 5 mm apical to CEJ. The protrusion was measured by the distance between the buccal surface of the tooth to the true vertical line established at 10 mm ahead of the T1 symphysis. Both T1 and T2 incisor anterior–posterior positions were measured to the T1 true vertical line to eliminate mandibular bony structure changes due to the normal growth and development. Teeth anteriorly positioned to the true vertical line was recorded with a positive value while posteriorly positioned to the line was recorded with a negative value. Over the time period, a positive value was indicative of protrusion while a negative value was indicative of retrusion.

The vertical position of the mandibular incisors was measured by the distance between the incisal edge and the true horizontal line established at the T1 inferior border of symphysis. Both T1 and T2 incisor vertical position were measured to the T1 true horizontal line to eliminate the difference of the inferior border of symphysis between T1 and T2 due to the normal growth and development of the mandible. Over the time period, a positive value was indicative of an eruption while a negative value was indicative of an intrusion.

All measurements were performed on both left and right sides of each sample. Thus, the sample size was 32 for the RME group and 36 for the RME + LB group. All the measurements were taken by the same examiner (O.G.), and 9 samples were randomly selected and remeasured at a 1-week interval to test the reliability and repeatability of the current measurement protocol. The interclass correlation coefficient (ICC) of each parameter was calculated utilizing the IBM SPSS software (Statistical Package for Social Sciences version 26.0, Chicago, IL, USA). Due to the large range of treatment times and the considerable difference in the time ranges between T1 and T2 of the samples included in the study, it was determined that statistics should be evaluated on both total changes as well as an average change per year. In doing so, the authors hoped to eliminate skewing of the results that may occur from a patient undergoing the expected changes from growth over a longer T1 to T2 time period. The Shapiro–Wilk normality test was performed using OriginPro 8 (Origin Lab Corp., Northampton, MA, USA). Some data did not follow normal distribution so all data are presented as raw data overlapped with a Median ± 95% confidence interval. For comparisons between different timepoints within a group, Wilcoxon matched-pairs signed rank test was used. For intergroup comparisons, Mann–Whitney *U* test was used. For all data presented in this manuscript, *p* < 0.05 (*) was considered as a statistically significant difference.

## 3. Results

### 3.1. Patient Demographic Information Comparison between Groups

For the sample population included in the current study, there was no significant difference in T1 age, T2 age and time intervals between T1 and T2 (Table 1). There was also no significant difference in gender distribution of the samples enrolled in each group (Table 1). This supports the comparability of the two groups.

For all the measurements, the lowest ICC was 0.922, which suggests high consistency and reliability of the current measurement protocol (Table 2).

### 3.2. Mandibular Central Incisor Inclination Change

**Figure 2:**
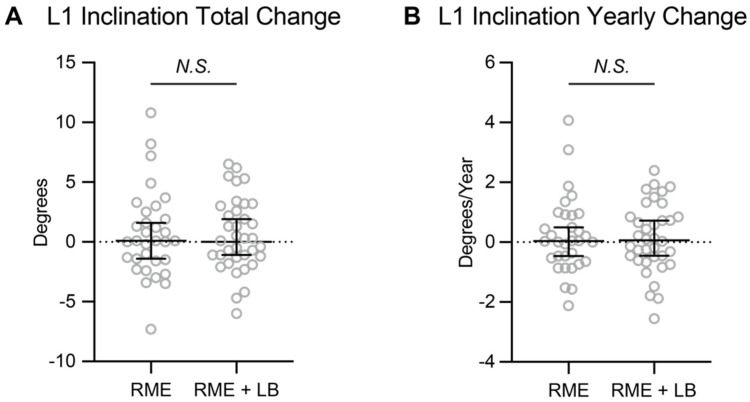
The (**A**) total change and (**B**) yearly change of the mandibular central incisors’ inclination. The data are presented as raw data overlapped with Median ± 95% confidence interval. RME: rapid maxillary expansion; LB: lip bumper; *N.S.*: not significant.

The majority of the previous studies concluded that significant amounts of proclination of the mandibular central incisors was observed after lip bumper therapy. In the current study, both RME and RME + LB groups did not show significant change in mandibular incisor inclination when comparing T2 image with T1 image (Table 3). In addition, when comparing between groups, there was no statistically significant differences in total changes or yearly changes of the mandibular central incisor inclination (Table 3, Figure 2). Thus, in the current study, the lip bumper did not cause proclination of mandibular central incisors.

### 3.3. Mandibular Central Incisor Anterior–Posterior Position Change

As some of the previous studies indicated, lip bumper treatment can cause protrusion of the mandibular central incisors. To further investigate, the anterior–posterior position of the mandibular central incisors was evaluated at three different vertical levels.

**Figure 3:**
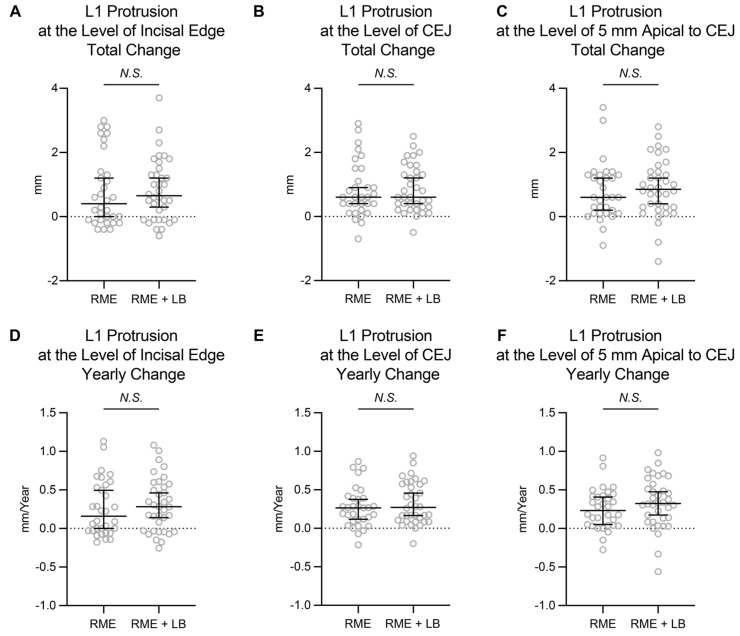
The total change and yearly change of the mandibular central incisors anterior–posterior position. The data are presented as raw data overlapped with Median ± 95% confidence interval. CEJ: cementoenamel junction; RME: rapid maxillary expansion; LB: lip bumper; *N.S.*: not significant.

When comparing the T1 and T2 images, significant amounts of protrusion were detected in the RME + LB group at all three evaluation levels (Table 3). However, the amount of protrusion in the RME + LB group was not statistically significantly different than that in the RME group (Table 3, Figure 3). Thus, the amount of mandibular incisor protrusion observed in the RME + lip bumper groups may be from normal mandibular dentition growth and development instead of from treatment effect.

### 3.4. Mandibular Central Incisor Vertical Position Change

Lastly, we evaluated if the lip bumper caused a change in the vertical position of the mandibular central incisors.

**Figure 4:**
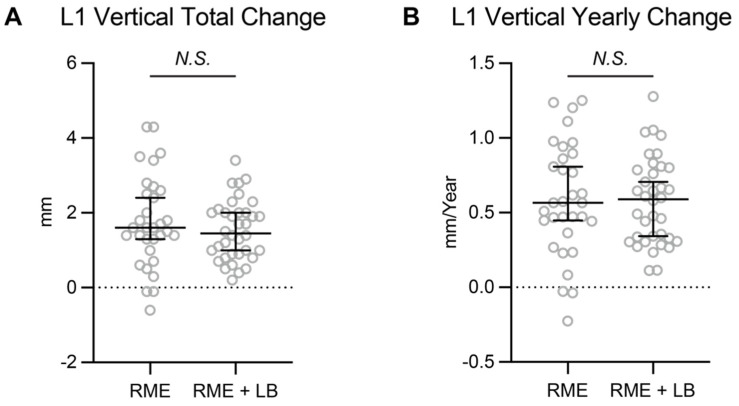
The (**A**) total change and (**B**) yearly change of the mandibular central incisors’ vertical position. The data are presented as raw data overlapped with Median ± 95% confidence interval. RME: rapid maxillary expansion; LB: lip bumper; *N.S.*: not significant.

As shown in Table 3, both RME and RME + LB groups showed statistically significant amounts of mandibular central incisor eruption during the phase I treatment. When comparing between these two groups, the amount of change was not statistically significantly different (Table 3, Figure 4). Thus, the amount of mandibular incisor eruption observed in the RME + lip bumper group may be from normal mandibular dentoalveolar growth and development instead of from treatment effect.

## 4. Discussion

In the current study, a longitudinal CBCT database was utilized to evaluate the mandibular incisors’ positional changes introduced by lip bumper therapy. This question has been previously evaluated and published in the literature utilizing lateral cephalograms. The novelty of the study comes from the use of CBCT images, which decreases magnification and distortion. Additionally, by utilizing 3D mandibular superimposition, discrepancies in measurements due to a tracing error between timepoints was reduced. Furthermore, by comparing with the control group, the study eliminated the effects of normal mandibular dentition growth and development.

Three positional changes were evaluated in this study: changes in inclination, changes in protrusion and vertical development of the mandibular incisors. Firstly, the central incisor inclination according to our study had no significant difference between the experimental group (RME + LB group) and the control group (RME group) in terms of total or annual change. This finding is contrary to numerous prior studies including O’Donnell et al., Grossen et al. and Davidovitch et al., who all found at least 2.5° of lower incisor proclination from lip bumper treatment [5,6,7]. On the other hand, the result of our study agreed with the study from Raucci et al. [9]. There are several possible explanations for this finding in our study. The first explanation is that the use of CBCT allowed for a more reliable measurement since there is no overlap of other anatomical structures over the lower incisor root [17]. Another explanation is that there were no control groups in either the O’Donnell et al. or Grossen et al. studies. Therefore, there is no way to determine if the proclination seen was just a normal part of growth and development in their subjects. Thirdly, the mandibular border has active modeling and remodeling during growth and development. Thus, using the changes of the incisal mandibular plane angle (IMPA) might not be a reliable parameter to evaluate the treatment effects. The most likely explanation for our finding of no change in lower incisor inclination compared to previously documented findings is that our study window was large, since the T2 image was not taken immediately after the lip bumper therapy. In the current study, the median time interval between lip bumper removal and T2 images was about 1.5 months. Thus, any treatment effect of incisor proclination introduced by the lip bumper had already relapsed and could not last until the timepoint when patients were ready for phase II comprehensive treatment.

Secondly, the anterior–posterior position change of the mandibular incisors was evaluated. About 0.6 mm of mandibular incisor protrusion was observed in the RME + LB group. This finding is different than the Jacob study, which found the lower incisor tip protruded 1.2 mm, and the O’Donnell study, which found a protrusion of 0.9 mm [5,8]. However, our findings were more closely similar to a different study which found the mandibular incisors protruded approximately 0.5 mm following lip bumper treatment [6]. When comparing the amount of mandibular incisor protrusion between RME and RME + LB groups, no statistically significant difference was detected. It is worth noting that the yearly change of mandibular incisor protrusion observed in the current study (0.16 mm/yr at the incisal edge of the RME group, 0.28 mm/year at the incisal edge of the RME + LB group) is close to the estimated yearly change (0.3 mm/year) of normal growth and development provided by Buschang et al. [19]. Overall, the current study indicated that the protrusion observed during the phase I treatment was entirely the result of normal mandibular dentition growth and development.

Another measurement evaluated in this study was the vertical eruption of the lower incisors. There was a statistically significant difference in the vertical position between T1 and T2 for both groups, but there was no statistically significant difference between groups. The total amount of vertical changes in our study (1.60 mm in the RME group, 1.45 mm in the RME + LB group) was similar to a previously reported study on lip bumper treatment that found eruption to be approximately 1.5 mm [8]. However, the previously reported study lacked a control group [8]. Thus, it is concluded that lip bumper therapy does not have a significant effect on vertical development of the lower incisors.

This study has multiple limitations that need to be discussed before accepting conclusions and interpreting the data’s clinical relevance. The study was performed retrospectively on previously treated subjects. Due to the nature of the subjects, random allocation of treatment groups could not be performed. In addition, because of the retrospective nature of the data the exact delivery, the adjustment and design of the lip bumper was not controlled in the study. Furthermore, the length of lip bumper treatment varied within the experimental group. Most of the prior literature on the dentoalveolar effects of the lip bumper had treatment times not greater than one year. As previously cited in Murphy et al., the majority of treatment effects of lip bumper therapy are seen within the first 300 days [20]. In addition, as previously discussed there was a large variance in the total treatment time within groups and between the groups. Due to the age of the patient population, a significant amount of growth and development was expected to occur during the treatment period. Even though there were no statistically significant difference on T1 age, T2 age and the time interval between the two groups, the large variance in the length of total treatment of each individual could distort the results. Besides, atypical swallowing patterns have been proven to affect the upper and lower incisors’ proclination in children and adolescents [21]. In the current study, we did not evaluate and compare the swallowing patterns of the involved subjects between the two groups. Future studies should try to have control and experimental groups with similar intervals between data timepoints, and consider the potential influence of atypical swallowing patterns. Finally, although the CBCT images provide the unique possibility to evaluate a 3D anatomic structure without the interference of overlapping structures, there are some detected differences within or between the groups that are near one voxel size and therefore, near the detection limit of the method used.

## 5. Conclusions

In the present study, the lip bumper did not cause significant proclination, protrusion or extrusion of the mandibular incisors. The dentoalveolar effects of lip bumper therapy need further evaluation with a well-controlled, prospective CBCT study in order to guide future treatment methodologies.

## Figures and Tables

**Table 1:** The demographic information of the two groups. RME: rapid maxillary expansion; LB: lip bumper; F: female; M: male; yrs: years. The Chi-square test was performed for the gender distribution comparison between groups. The Mann–Whitney *U* test was performed for the age and time interval comparisons between groups.

	RME Group	RME + LB Group	*p*-Value
Patient Number	16 (9 F, 7 M)	18 (13 F, 5 M)	0.3307
Age at T1 (yrs, median [Min, Max])	8.86 [7.62, 10.48]	9.46 [8.29, 10.29]	0.0581
Age at T2 (yrs, median [Min, Max])	11.82 [10.81, 13.82]	12.10 [10.98, 12.99]	0.0928
Time Interval (yrs, median [Min, Max])	2.98 [2.11, 3.84]	2.62 [1.75, 3.66]	0.2772

**Table 2:** The Interclass Correlation test results of each parameter.

	ICC (Absolute Agreement)	
	Interclass Correlation	95% Confidence Interval [Lower Bound, Upper Bound]
L1 Inclination (∘)	0.989	[0.978, 0.994]
L1 Protrusion-Incisal Edge (mm)	0.922	[0.983, 0.996]
L1 Protrusion-CEJ (mm)	0.991	[0.975, 0.996]
L1 Protrusion-CEJ5 (mm)	0.973	[0.948. 0.986]
L1 Vertical (mm)	0.994	[0.989, 0.997]

**Table 3:** The amount of total changes of each measurement parameters for both groups. For T1 vs. T2 comparisons within group, Wilcoxon matched-pairs singed rank test was used. For RME vs. RME + LB comparisons, Mann–Whitney *U* test was used.

	Total Change				
	RME Group		RME + LB Group		RME vs. RME + LB *p*-Value
	T2-T1 Changes (Median [Min, Max])	T1 vs. T2 *p*-Value	T2-T1 Changes (Median [Min, Max])	T1 vs. T2 *p*-Value	
L1 Inclination (∘)	0.10 [−7.30, 10.80]	0.5732	0.20 [−6.00, 6.50]	0.3644	0.8142
L1 Protrusion-Incisal Edge (mm)	0.40 [−0.40, 3.00]	0.0006	0.65 [−0.60, 3.70]	<0.0001	0.6312
L1 Protrusion-CEJ (mm)	0.60 [−0.70, 2.90]	<0.0001	0.60 [−0.50, 3.00]	<0.0001	0.7433
L1 Protrusion-CEJ5 (mm)	0.60 [−0.90, 3.40]	<0.0001	0.85 [−1.40, 2.80]	<0.0001	0.4138
L1 Vertical (mm)	1.60 [−0.60, 4.20]	<0.0001	1.45 [0.20, 3.40]	<0.0001	0.5349

## Data Availability

The data presented in this study are contained within this article.
